# Xyloglucan endotransglucosylase/hydrolase 25 positively regulates the lead tolerance in *Raphanus sativus*

**DOI:** 10.3389/fpls.2025.1607751

**Published:** 2025-06-13

**Authors:** Tong Han, Yongmei Cui, Yang Jing, Meiying Liu, Xuanchao Chen, Yinghui Song, Xinping Gu, Jiahao Wang, Long Wang

**Affiliations:** ^1^ Shandong Provincial Key Laboratory of Biochemistry and Molecular Biology, School of Advanced Agricultural Sciences, Weifang University, Weifang, China; ^2^ Laboratory for Research and Utilization of Qinghai Tibet Plateau Germplasm Resources, Agriculture and Forestry Academy, Qinghai University, Xining, China; ^3^ Technology Development Department, Shouguang Vegetable Industry Holding Group, Weifang, China

**Keywords:** Raphanus sativus, XTH, lead stress, transcription factor, gene family

## Abstract

*Raphanus sativus*, an important root vegetable native to China, is widely cultivated for its nutritional value and diverse applications. However, it is highly sensitive to lead (Pb) stress, with Pb predominantly accumulating in the roots. Previous studies have highlighted the pivotal role of xyloglucan endotransglucosylase/hydrolase (XTH) in plant responses to heavy metal stress. Despite this, a comprehensive identification, molecular characterization, and functional analysis of the *XTH* gene family in *R. sativus* has been lacking. In this study, 28 *XTH* genes were identified in *R. sativus*. Gene structure analysis revealed the presence of eight conserved motifs, along with variations in exon-intron organization and chromosomal distribution across all chromosomes. Phylogenetic analysis of *XTH* genes from *R. sativus*, *Arabidopsis thaliana* and *Oryza sativa* grouped them into five distinct clades, suggesting their evolutionary conservation and potential functional diversification. Transcriptome sequencing and qRT-PCR analysis showed that *RsXTH25* was strongly induced by Pb stress. Transgenic hairy roots overexpressing *RsXTH25* exhibited enhanced Pb tolerance, evidenced by reduced chlorosis, increased fresh weight, improved photosynthetic performance, and lower oxidative damage under Pb stress. Furthermore, several transcription factors, such as RsERF2, RsHD-ZIP22, and etc., exhibited strong positive correlations with *RsXTH25*, implying their roles in regulating Pb-induced *RsXTH25* expression. Overall, this study provides insights into the *XTH* gene family in *R. sativus* and highlights their critical roles in Pb stress response.

## 1 Introduction

Lead (Pb), a heavy metal element widely present in the environment, does not contribute to the structural composition of plant tissues or participate in cellular metabolic activities (Carocci et al., 2016; Henry et al., 2015). However, its excessive accumulation can exert severe toxic effects on plants. In agriculture, soil Pb pollution can degrade soil fertility, consequently reducing crop yields (Aslam et al., 2021; Yan et al., 2023a). Moreover, environmental Pb is readily absorbed by plants through their roots and leaf surfaces, leading to its accumulation and distribution in various plant tissues. This can result in agricultural products exceeding permissible Pb limits, thereby compromising product quality and safety (Collin et al., 2022; Levin et al., 2021; Sun and Luo, 2018). Plants have evolved various mechanisms to resist lead (Pb) stress, with the cell wall playing a crucial role as the first line of defense (Collin et al., 2022). As a major structural component, the cell wall can act as a barrier to restrict Pb entry into cells by immobilizing Pb ions through adsorption onto its components, such as pectin, cellulose, hemicellulose, and lignin (Wu et al., 2020). Among the components of the cell wall, hemicellulose, plays a crucial role in responding to Pb stress by binding Pb ions, thereby immobilizing them and limiting their toxicity to plant cells (Zhang et al., 2020b).

Xyloglucan (XyG) is an essential hemicellulose polymer in the primary cell walls of dicotyledonous plants and non-commelinid monocotyledonous plants (Hrmova et al., 2022; Yan et al., 2019). Numerous genes are involved in the biosynthesis of xyloglucan, including those encoding glycosyltransferases that assemble its β-1,4-glucan backbone and side-chain modifications (Hayashi and Kaida, 2011; Pauly et al., 1999; Pauly and Keegstra, 2016). Among these enzymes, xyloglucan endotransglucosylase/hydrolase (XTH) enzymes play a key role in modifying xyloglucan by cleaving and reconnecting its chains, thereby regulating cell wall remodeling and expansion (Julian and Zabotina, 2022; Stratilová et al., 2020; Suo et al., 2024). To date, the *XTH* gene family has been identified across numerous plant species, with the number of *XTH* genes varying significantly among them. In monocotyledonous plants, rice, wheat, and pineapple contain 29, 71, and 24 *XTH* genes, respectively (Han et al., 2023; Li et al., 2019; Yokoyama et al., 2004). In contrast, dicotyledonous plants such as *Arabidopsis*, poplar, and sweet potato have 33, 38, and 36 *XTH* genes, respectively (Yang et al., 2023; Yokoyama and Nishitani, 2001; Zhang et al., 2023). In *Arabidopsis*, Pb stress obviously induces the expression of *XTH18*, and mutation of *XTH18* enhanced the tolerance of plant to Pb stress (Zheng et al., 2021). In addition, the expression of *XTH5*, *XTH20*, and *XTH31* are rapidly and consistently upregulated in *Arabidopsis* roots under Pb stress. This imply that XTHs might play a crucial role in plant response to Pb stress (Zheng et al., 2021). However, the role of *XTH* genes in response to Pb stress in other plant species is still poorly understood.

*R. sativus*, a widely cultivated root vegetable of the Brassicaceae family, is propagated through seeds and valued for its nutritional and medicinal properties. *R. sativus* roots are abundant in bioactive compounds, including glucosinolates, anthocyanins, and antioxidants, which exhibit anti-inflammatory, anticancer, and antioxidant activities, underscoring their significant economic and health benefits (Gamba et al., 2021; Khan et al., 2022). However, *R. sativus* is highly sensitive to lead (Pb) stress, which disrupts root system development, impairs water and nutrient uptake, and induces oxidative cellular damage. These effects lead to stunted growth, reduced yields, and economic losses, posing a challenge to sustainable production (Tang et al., 2021; Wang et al., 2015, 2013). Despite the known impacts of Pb stress, the molecular mechanisms underlying *R. sativus* responses to this heavy metal remain poorly understood.

To date, the identification of the XTH family in *R. sativus* under lead (Pb) stress remains largely unexplored. In this study, we first analyzed the homologous evolution and protein domains within the *XTH* gene family. Subsequently, we observed a significant upregulation of *RsXTH25* in *R. sativus* roots under Pb stress conditions. Functional analysis of RsXTH25 further revealed its role in mediating Pb stress tolerance. In addition, several transcription factors, such as *RsERF2*, *RsHD-ZIP22*, and etc., exhibited strong positive correlations with *RsXTH25*, implying their roles in regulating Pb-induced *RsXTH25* expression. These findings provide a foundational basis for future research into the molecular mechanisms of Pb stress responses in *R. sativus* and the development of genetically improved *R. sativus* varieties with enhanced Pb tolerance.

## 2 Materials and methods

### 2.1 Plant materials

The seeds of cherry *R. sativus* inbred line were disinfected, washed and germinated for 3 days, and then cultured for 3 weeks under the photoperiod of 24°C light for 14 hours and 20°C darkness for 10 hours. Transfer seedlings of similar size to plastic containers filled with Hoagland nutrient solution. After one week, the plants were treated with 0 and 200 mg/L Pb(NO3)_2_. Samples are taken every 4 days. Seedlings grown in Pb-free nutrient solution were used as control. Three replicates were used for each treatment, each with 6-8 plants.

### 2.2 Identification of XTH genes in R. sativus

To identify *XTH* genes in *R. sativus*, genomic nucleotide and amino acids sequences were obtained from the *R. sativus* genome database. HMMER (version 3.4) was used to screen for sequences containing both the Glycosyl Hydrolase domain (PF00722) and the Xyloglucan Endotransglucosylase (XET) C-terminal domain (PF06955), as annotated in the Pfam database (https://pfam.xfam.org/). The significance threshold for this screening was set at P < e^–5^. This analysis yielded 41 candidate protein. Subsequent domain confirmation using NCBI-CDD identified 28 putative *XTH* genes in the *R. sativus* genome (Mukherjee et al., 2009).

### 2.3 Phylogenetic analysis of XTH gene family

Full-length amino acid sequences XTHs of *A. thaliana* and *O. sativa* were retrieved from the Phytozome database and aligned with *R. sativus XTH* genes using MAFFT (version 7.526) (Katoh et al., 2019). A maximum likelihood phylogenetic tree was constructed with IQ-TREE (version 2.2.2.7) using default parameters and 1000 bootstrap replicates. The resulting tree was visualized and edited using Evolview v2 (He et al., 2016).

### 2.4 Gene structure analysis of XTH gene family in R. sativus

Conserved motifs in XTH proteins were identified using the MEME (version 5.5.4) Suite. These motifs were then validated against the NCBI-CDD to confirm their biological relevance. Gene structures and chromosomal locations were determined using the GFF3 annotation file from the *R. sativus* genome and visualized with TBtools II (version 2.016) (Chen et al., 2023).

### 2.5 RNA extraction and sequencing

Total RNA was extracted from plant tissue as described in (Liu et al., 2024). RNA quality was assessed with a NanoDrop 2000 spectrophotometer. High-quality RNA samples were then performed for next-generation sequencing using the Illumina platform. Raw sequencing data were subjected to quality control, during which reads containing adapters, poly-N sequences, or low-quality regions were removed. The Q20 and Q30 scores were evaluated using FastQC to further assess data quality. Clean reads were subsequently aligned to the *R. sativus* reference genome using HISAT2. Gene expression levels were quantified with featureCounts and normalized using the fragments per kilobase of transcript per million mapped reads (FPKM) method. The criteria for filtering differentially expressed genes were |log2FC| ≥ 1 and FDR < 0.05.

### 2.6 Reverse transcription and qRT-PCR analysis

Total RNA was extracted from *R. sativus* samples using the FastPure Plant Total RNA Isolation Kit (Vazyme, China) (Yan et al., 2024). High-quality RNA was used for cDNA synthesis, then used to perform qRT-PCR as described in (Liu et al., 2023). Relative gene expression was calculated using the 2^^–ΔΔCT^ method with *RsActin* as the reference gene (Yan et al., 2023b).

### 2.7 Transformation of hairy roots in R. sativus

The transformation of hairy roots in *R. sativus* was described in (Qin et al., 2024). In brief, the *Agrobacterium rhizogenes* strain MSU440 harboring the recombinant plasmid pCAMBIA1300, stored at -80°C, was revived in LB liquid medium containing 100 mg/L kanamycin and 50 mg/L streptomycin. The culture was incubated until the optical density at 600 nm (OD_600_) reached 0.6. The pellet was collected and resuspended in half-strength MS liquid medium. Under aseptic conditions, rootless seedlings were immersed in the bacterial suspension (OD_600_ = 0.8) supplemented with 200 μM acetosyringone for 10 minutes. The infection was carried out at 28°C on a shaking incubator set to 220 rpm. Following infection, the seedlings were co-cultivated in darkness for 2 days. Subsequently, they were transferred to decontamination medium containing 500 mg/L cefotaxime and cultured in a growth chamber at 25°C under a 16 h light/8 h dark photoperiod. Upon root induction, the transformed seedlings were treated with 200 mg/L Pb(NO_3_)_2_ for 20 days, after which the growth status of the hairy roots was assessed.

### 2.8 Subcellular localization

The coding sequences (CDS) of *RsXTH25* were inserted into the pCAMBIA1300-GFP expression vector. The resulting fusion constructs were transiently expressed in tobacco leaf epidermal cells through *Agrobacterium tumefaciens*-mediated infiltration (Wang et al., 2024). At five days post-infiltration, GFP signals were observed using a confocal laser scanning microscope (LSM510, Carl Zeiss, Germany) following standard protocols.

### 2.9 Statistical analysis

All results are expressed as mean ± standard deviation (SD) from at least three replicates. The data were analyzed using one-way analysis of variance (ANOVA) in SPSS Statistics 29.0, with a P-value < 0.05 considered statistically significant.

## 3 Result

### 3.1 Identification of XTH gene family in R. sativus

In order to excavate the *XTH* (Xyloglucan Endotransglucosylase/Hydrolase) gene family in *R. sativus*, the Glycosyl hydrolases domain (PF00722) and the Xyloglucan endo-transglycosylase (XET) C-terminus domain (PF06955) were utilized to search for *XTH* genes via HMMER search. The results identified 45 and 41 genes respectively, among which 41 genes contained both domains. Further domain confirmation through NCBI-CDD revealed a total of 28 *XTH* genes in *R. sativus*.

### 3.2 Gene structure and conserved motif identification of XTH gene family in R. sativus

**Figure 1:**
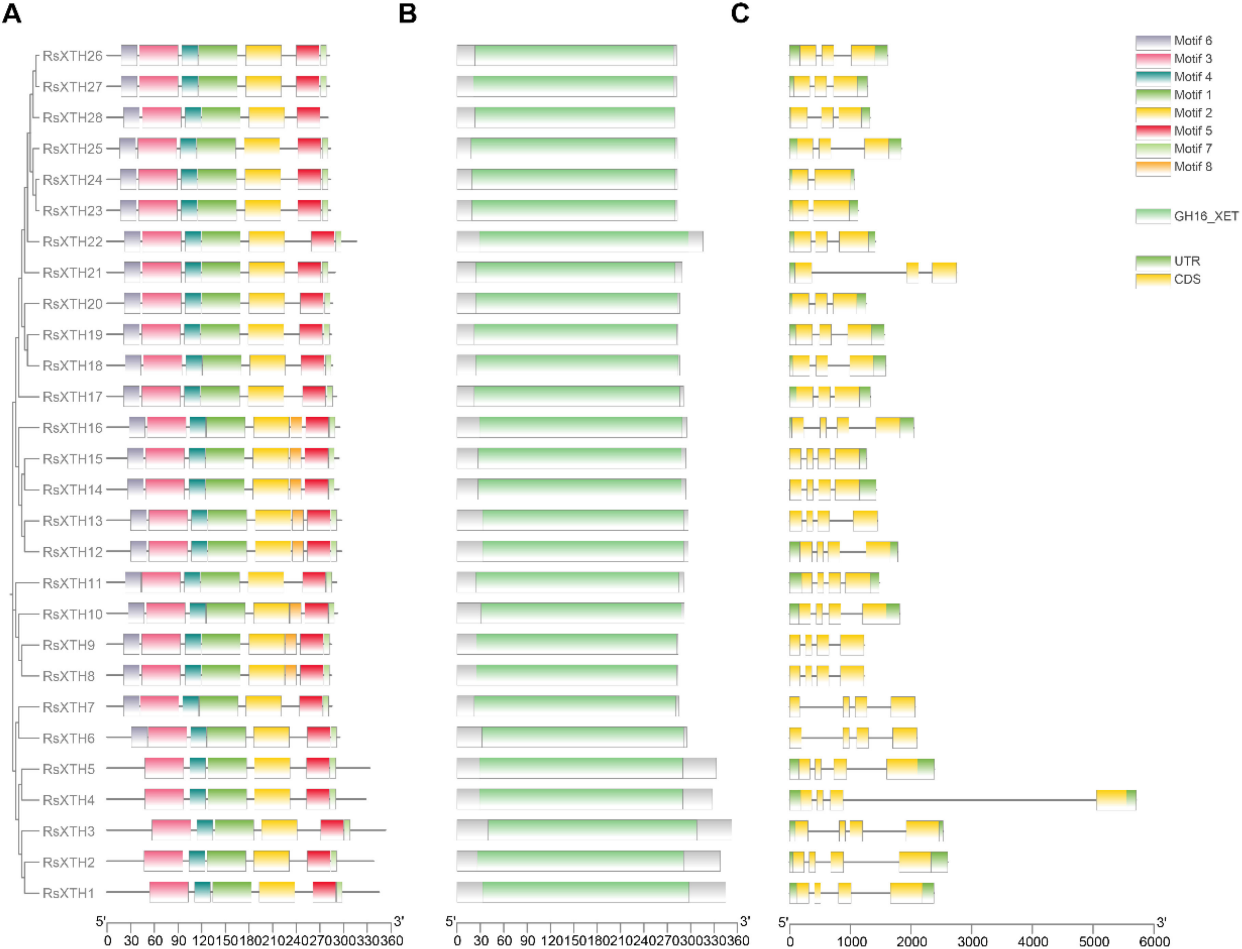
Phylogenetic relationship, and gene structure of *RsXTH* gene family. **(A)** The phylogenetic relationship and motif distribution of RsXTHs. **(B)** The conservative structural domains of RsXTHs. **(C)** The distribution of coding sequences in RsXTHs.

**Figure 2:**
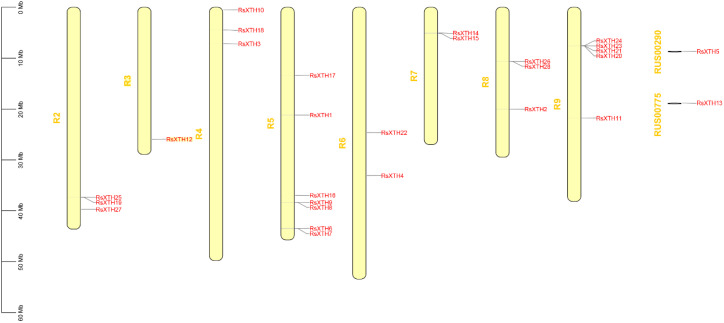
Chromosomal distribution of *RsXTHs* in *R. sativus*.

To further analyze the structural diversity of the *RsXTH* gene family, the conserved motifs were identified using MEME by TBtools-II. **Figure 1** illustrates that the 28 *XTH* genes collectively harbor 8 conserved motifs. Motif 1, Motif 2-5, and Motif 7 are ubiquitous across all *XTH* genes. Motif 6 is consistently present from *XTH6* to *XTH28*, whereas motif 8 is only found in a select few *XTH* genes. RsXTHs show a conserved structure, and the NCBI-CDD analysis notably reveals that all *R. sativus* XTHs possess the conserved XTH domain (GH16_XET), with an average amino acid length of 298 aa, a maximum of 353 aa, and a minimum of 280 aa. The *XTH* genes display structural diversity at the DNA level, with exon counts varying between 2 and 4, and intron counts ranging from 1 to 3. As shown in **Figure 2**, the chromosomal localization analysis indicates that XTHs are not only distributed across all chromosomes but also present on segments RUS00775 and RUS00290.

### 3.3 Phylogenetic analysis of XTH gene family

**Figure 3:**
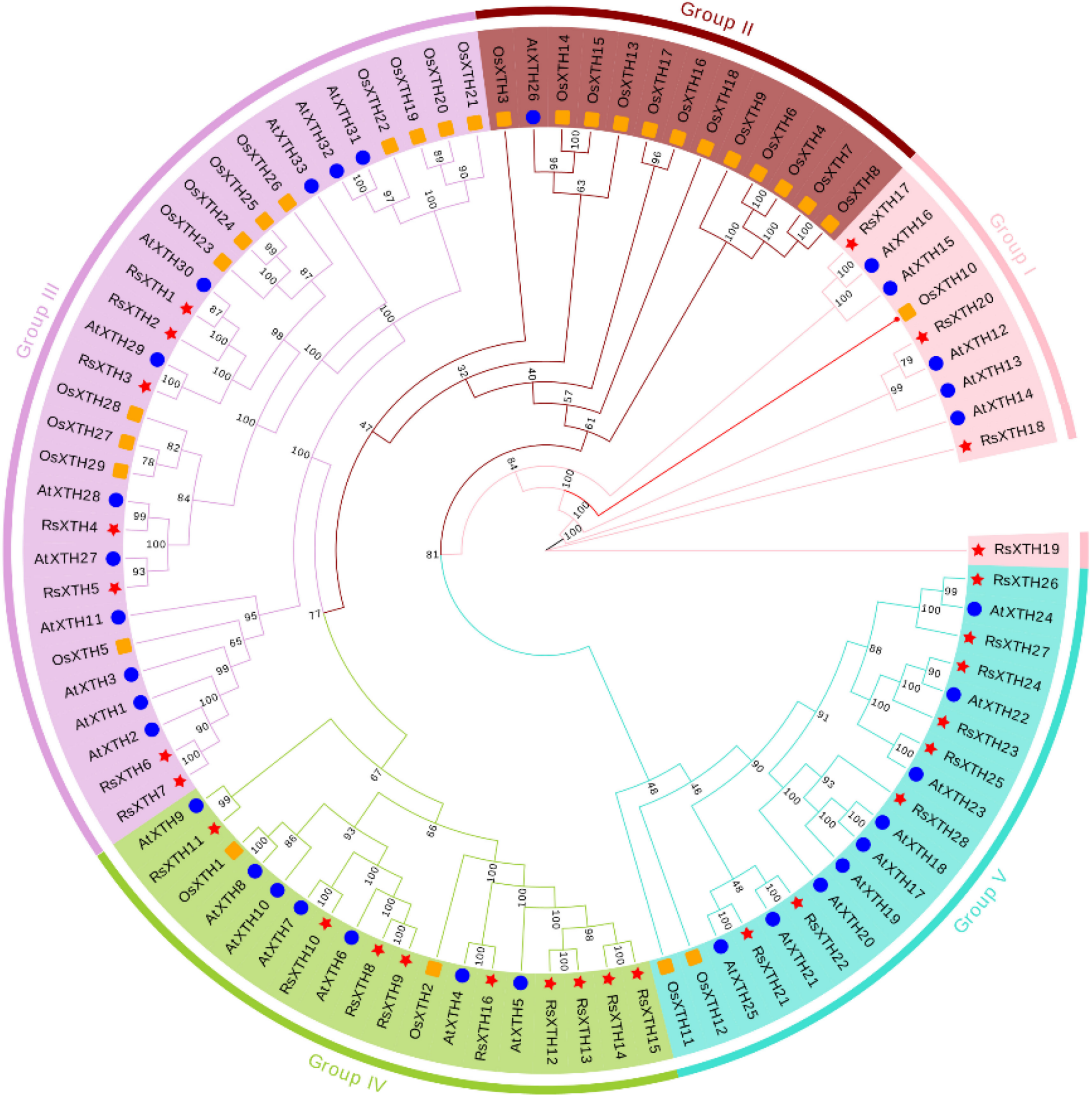
Transcriptome analysis of *R. sativus* under Pb treatments. **(A)** Principal component analysis (PCA). **(B-D)** The volcano plot of differential expression genes (DEGs) under Pb treatments. **(E)** The heatmap of *RsXTHs* under Pb treatments.

The full-length amino acid sequences of *XTH* genes from *R. sativus*, *A. thaliana* and *O. sativa* were aligned by MAFFT (**Supplementary Table S2**). Subsequently, a phylogenetic tree was constructed using IQ-TREE with the maximum likelihood estimate method. As illustrated in **Figure 3**, all *XTH* genes can be categorized into five distinct groups. Notably, Cluster 3 and Cluster 5 contain the highest number of *XTH* genes, with a high degree of sequence similarity among the genes within each cluster. Further statistical analysis reveals that *XTH* genes from *R. sativus* and *A. thaliana* are almost distributed across other branches. In contrast, the XTH genes of *O. sativa*, a monocotyledonous plant, show a significant clustering in cluster 2. This finding highlights the widespread presence and potential functional diversity of the *XTH* gene family in monocotyledonous and dicotyledonous plants.

### 3.4 Transcriptome analysis of R. sativus seedlings under Pb treatment

**Figure 4:**
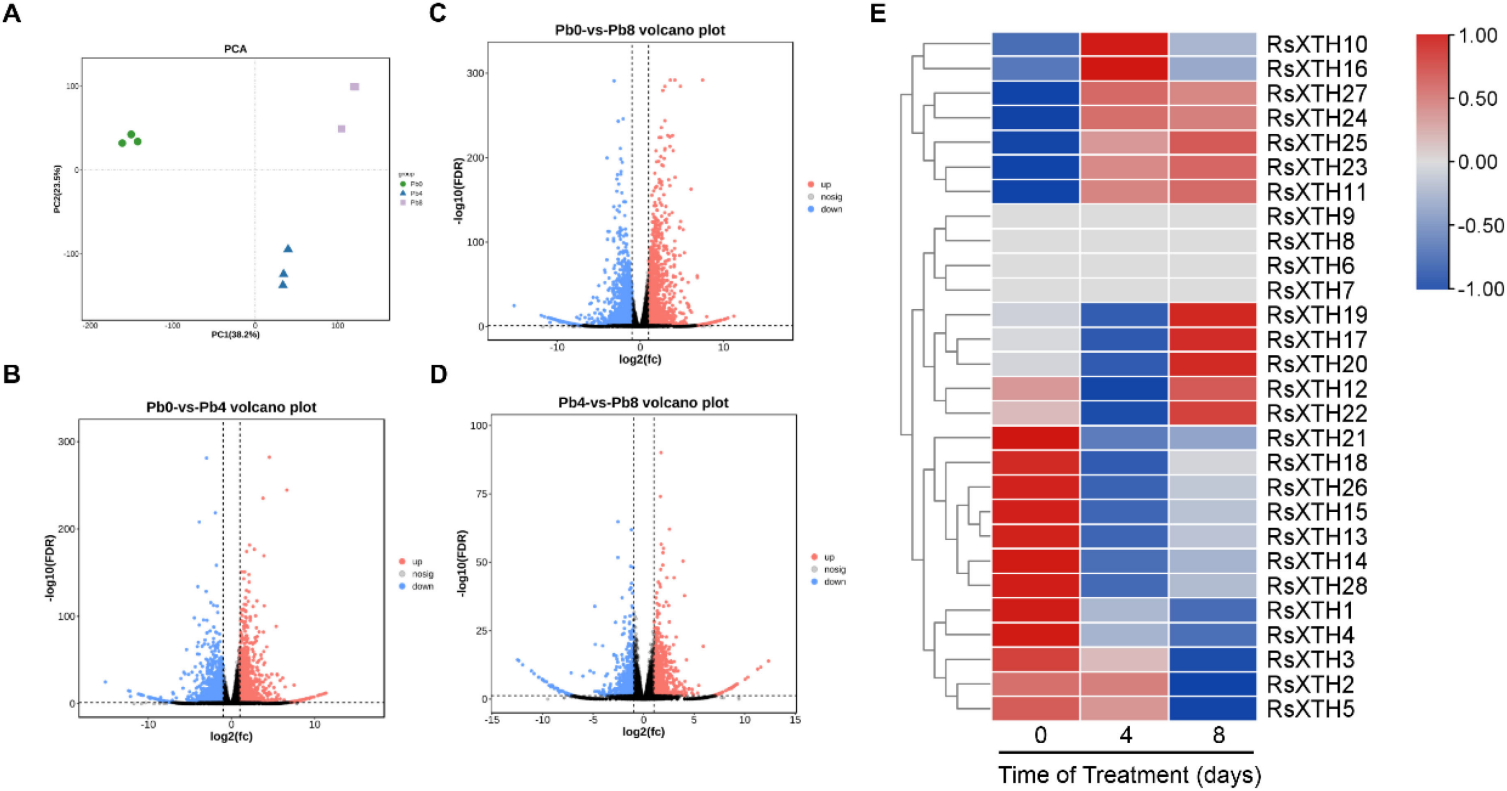
Quantitative RT-PCR analysis of *RsXTHs* under Pb treatments. The expression of *RsXTH11 RsXTH23*, *RsXTH24*, *RsXTH25*, and *RsXTH27* in *R. sativus* roots exposed to Pb stress was detected using qRT-PCR. Error bars indicate standard deviation (SD, n = 3). Different letters indicate significant differences according to one-way ANOVA (P < 0.05).

To explore the function of RsXTHs in *R. sativus* seedlings under Pb stress, transcriptome sequencing was conducted on seedlings exposed to a Pb concentration of 200 mg/L. The transcriptome data yielded high-quality results, as shown in **Supplementary Table S3**. Principal component analysis (PCA) revealed a close correlation among the three replicates for each sample, confirming the data’s reliability (**Figure 4A**). Through transcriptomic differential expressed genes (DEGs) analysis, a series of genes showing differences were found under different Pb treatment conditions (**Figures 4B-D**; **Supplementary Tables S4**, **S5**). Further expression analysis of *RsXTHs* under Pb stress showed that several *RsXTH* genes, including *RsXTH10*, *RsXTH11*, *RsXTH16*, *RsXTH27*, *RsXTH23*, *RsXTH24*, and *RsXTH25*, were significantly upregulated in response to Pb treatment. In contrast, *RsXTH1*, *RsXTH4*, *RsXTH3*, *RsXTH2*, and *RsXTH5* exhibited strong downregulation. Some genes, such as *RsXTH6*, *RsXTH7*, and *RsXTH9*, maintained relatively stable expression levels, suggesting minimal responsiveness to Pb stress (**Figure 4E**).

**Figure 5:**
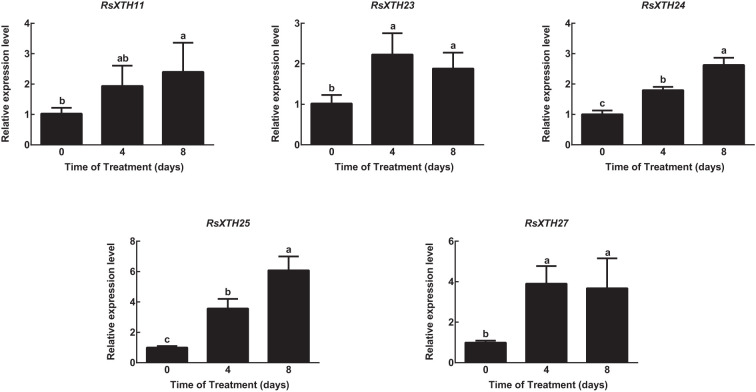
Effect of RsXTH25 on Pb tolerance in *R. sativus*. **(A)** Transcript levels of *RsXTH25* in the hairy roots of *OE-RsXTH25* transgenic plants. **(B)** Phenotypic analysis of *R. sativus* seedlings under Pb treatment. Scale bar = 1 cm. **(C–I)** Quantitative analysis of shoot fresh weight **(C)**, root fresh weight **(D)**, chlorophyll a content **(E)**, chlorophyll b content **(F)**, total chlorophyll content **(G)**, malondialdehyde (MDA) content **(H)**, and electrolyte leakage rate **(I)** in transgenic and wild-type roots under Pb treatment. Error bars in **(A)** and **(C–I)** represent standard deviation (SD, n = 3). Different letters indicate statistically significant differences as determined by one-way ANOVA (P < 0.05). Capital letters denote comparisons among control groups, while lowercase letters denote comparisons among Pb-treated groups.

To further validate the transcriptome results, qRT-PCR analysis was conducted on five *RsXTH* genes. As shown in **Figure 5**, the expression levels of *RsXTH11*, *RsXTH23*, *RsXTH24*, *RsXTH25*, and *RsXTH27*, were significantly upregulated under Pb treatment. Among them, *RsXTH25* exhibited the most substantial increase, with transcript levels peaking at day 8. These expression patterns were consistent with the RNA-seq data.

### 3.5 Transient overexpression of RsXTH25 enhances the tolerance to Pb stress in R. sativus

**Figure 6:**
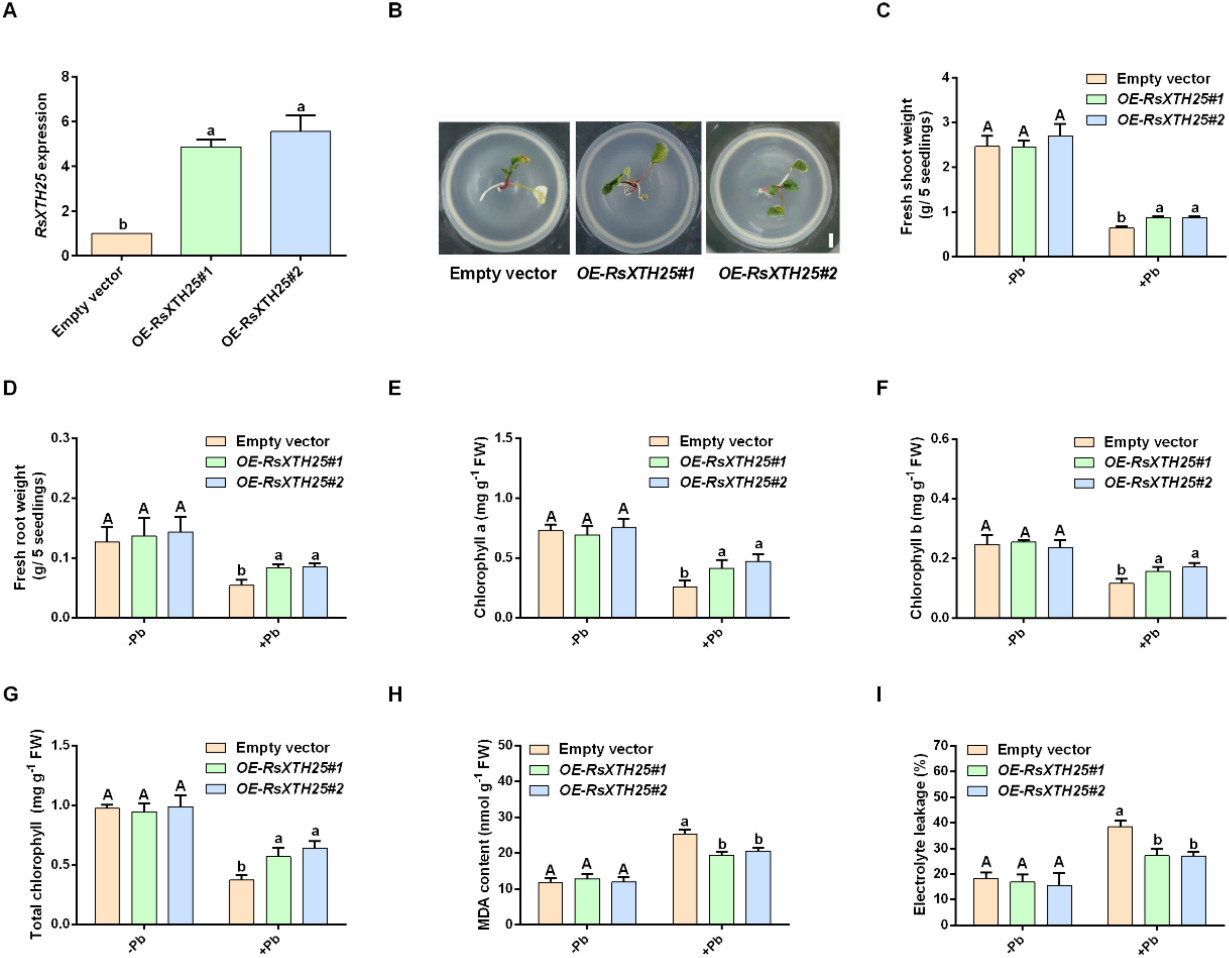
Subcellular localization of RsXTH25. Confocal microscopy images of *N. benthamiana* epidermal cells co-expressing the RsXTH25-GFP fusion protein and the plasma membrane marker pm-rk. Scale bars = 20 μm.

Given that *RsXTH25* was consistently induced by Pb stress and exhibited the highest fold change, we selected it for further functional analysis. To validate the role of RsXTH25 in regulating Pb tolerance in *R. sativus*, we generated transgenic hairy roots by infecting *R. sativus* seedlings with *Agrobacterium rhizogenes*. The transcript levels of *RsXTH25* in the hairy roots of two *OE-RsXTH25* lines were approximately fivefold higher than those in control plants transformed with the empty vector (**Figure 6A**). The fresh shoot and root weights of the transgenic lines were significantly higher than those of the wild type under Pb treatment (**Figures 6B-D**). To further evaluate Pb stress tolerance, we examined several physiological parameters. Transgenic seedlings maintained significantly higher levels of chlorophyll a, chlorophyll b, and total chlorophyll under Pb stress than wild-type plants (**Figures 6E-G**), indicating improved photosynthetic performance. Additionally, we assessed oxidative damage by measuring malondialdehyde (MDA) content and electrolyte leakage. As shown in **Figures 6H, I**, Pb exposure led to significant increases in both parameters across all lines; however, the increases were less pronounced in *OE-RsXTH25* lines than in wild-type plants. These findings suggest that transient overexpression of *RsXTH25* enhances Pb tolerance in *R. sativus* by alleviating Pb-induced oxidative damage and maintaining chlorophyll stability.

**Figure 7:**
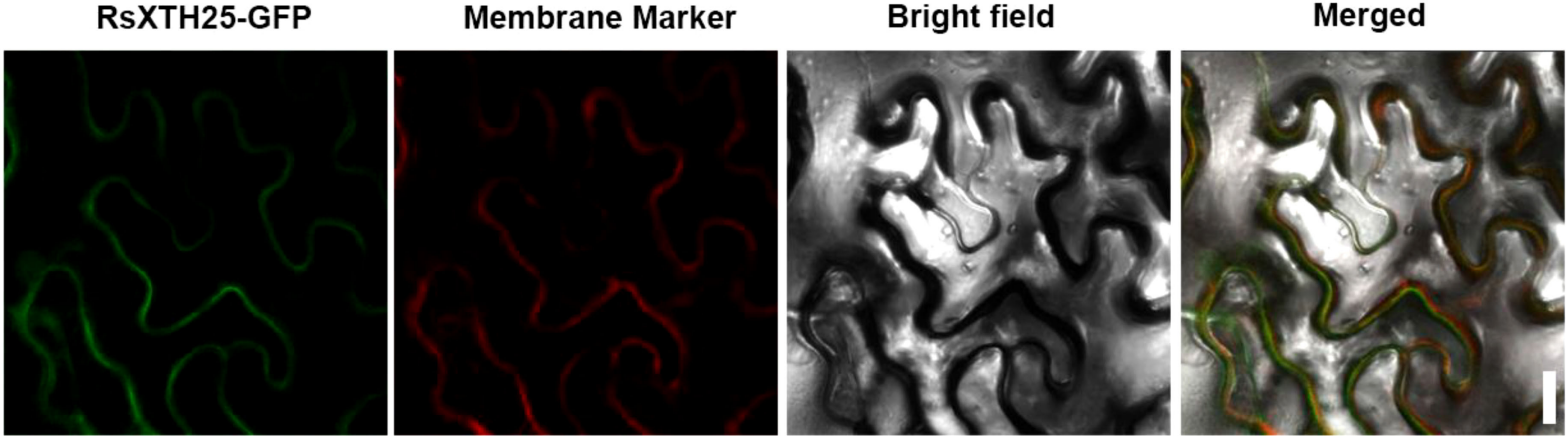
Upstream transcription factor analysis of *RsXTH25* under Pb Treatment. **(A)** Mfuzz clustering of the gene expression in *R. sativus* under Pb Treatment. **(B)** Classification of transcription factors in cluster 3. **(C)** Top 20 transcription factors positively correlated with RsXTH25 expression.

XTH proteins have been reported to localize to the endoplasmic reticulum, cell wall, or plasma membrane (Genovesi et al., 2008; Zhu et al., 2012). To gain insights into the functional role of RsXTH25, its subcellular localization was examined. As shown in **Figure 7**, the RsXTH25-GFP fusion protein was detected at the cell periphery, co-localizing with the plasma membrane marker pm-rk, indicating that RsXTH25 is localized to the plasma membrane.

### 3.6 Upstream transcription factor analysis of RsXTH25 under Pb treatment

**Figure 8:**
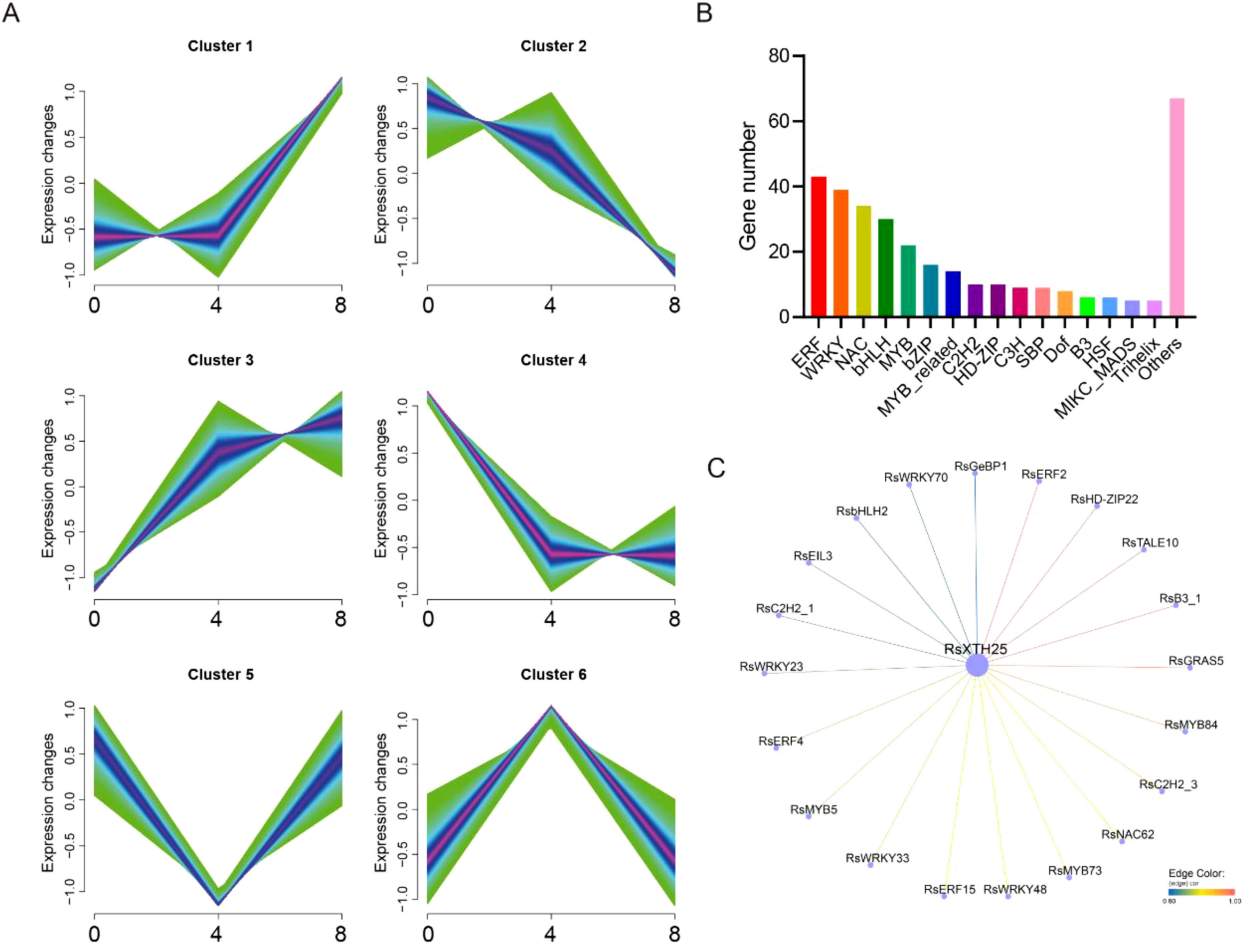
Phylogenetic relationships among XTH proteins in *R. sativus*, *A. thaliana* and *O. sativa.* The phylogenetic tree was investigated using the IQ-tree software. The RsXTHs were indicated with red stars, while the AtXTHs were indicated with blue circle and the OsXTHs were indicated with orange square.

To explore potential upstream regulators of *RsXTH25* in response to Pb stress, we conducted a comprehensive transcription factor analysis. Using mfuzz clustering analysis (**Figure 8A**), we identified that *RsXTH25* was grouped into cluster 3. Further analysis of all genes in this cluster revealed 333 transcription factors (**Figure 8B**), predominantly from the ERF, WRKY, NAC, and bHLH families, which are known to be involved in stress responses. To refine the candidate list, we performed Pearson correlation analysis to assess the expression relationships between these transcription factors and RsXTH25. As shown in **Figure 8C**, several transcription factors, including *RsERF2*, *RsHD-ZIP22*, *RsB3_1*, and *RsGRAS5*, exhibited strong positive correlations with *RsXTH25* and are therefore considered potential upstream regulators.

## 4 Discussion

Xyloglucan endotransglucosylase/hydrolase (XTH) is a key enzyme involved in the remodeling of plant cell walls, capable of cleaving and reconnecting xyloglucans (Eklöf and Brumer, 2010). Genome-wide analyses have identified varying numbers of *XTH* genes across different plant species, including 33 in *A. thaliana*, and 38 in poplar, showing its extensive involvement in various stages of the plant life cycle (Cheng et al., 2021; Ishida and Yokoyama, 2022; Yan et al., 2019). Therefore, studying the *XTH* gene family is of great significance for understanding plant growth and development. However, the comprehensive characterization of the *XTH* gene family in *R. sativus* remains elusive. Here, our study has unveiled the presence of 28 *XTHs* in *R. sativus.*

Phylogenetic tree illustrates the evolutionary relationships of the *XTH* gene family in *R. sativus* (*RsXTH*), *A. thaliana* (*AtXTH*) and *O. sativa* (*OsXTH*), clustering the genes into five distinct groups (Groups I–V) (**Figure 3**). The grouping reflects conserved evolutionary patterns, with notable gene expansion in *R. sativus*, likely due to segmental and tandem duplication events, as seen in other plant species such as soybean (Song et al., 2018). These duplications may have contributed to the diversification of *RsXTH* genes, enabling functional specialization. For example, genes within Group V (*RsXTH22*, *RsXTH24*, *RsXTH27*) or Group III (*RsXTH5*, *RsXTH6*, *RsXTH15*) could have undergone sub-functionalization or neofunctionalization, adapting to distinct biological roles. The clustering of *RsXTH* genes with *AtXTH* genes highlights conserved motifs and potential shared functions, such as regulating cell wall remodeling and environmental stress responses (Eklöf and Brumer, 2010; Hara et al., 2014). These findings provide a foundation for exploring the specific roles of *RsXTH* genes, especially under abiotic stress conditions like lead toxicity, and offer valuable insights into the evolution and functional diversity of the *XTH* gene family in *R. sativus*.

Plants can regulate their resistance to heavy metals such as Pb through structural modifications of the cell wall (e.g., polysaccharides, pectin, cellulose, lignin, and hemicellulose) and localized reinforcement (Krzesłowska, 2011; Krzesłowska et al., 2016). When plants are exposed to Pb stress, numerous cell wall-related genes are either upregulated or downregulated, indicating that cell wall modification serves as a common defense strategy for plants to adapt to Pb stress (Zheng et al., 2021). For example, in *A. thaliana*, the expression levels of *XTH5*, *XTH18*, *XTH20*, and *XTH31* were rapidly and consistently upregulated in response to Pb stress (Zheng et al., 2021). In Chinese cabbage, *BrXTH9_1* and *BrXTH16* were both upregulated under Pb stress in varieties with low (LPA) and high (HPA) Pb accumulation. Interestingly, *BrXTH9_1* showed a stronger induction in the HPA variety, whereas *BrXTH16* exhibited a more pronounced upregulation in the LPA variety (Gao et al., 2023). This suggests that different XTH members may have similar or distinct functions in responding to Pb stress, even within the same species or across different species. Here, we found that several *RsXTH* genes (*RsXTH10*, *RsXTH11*, *RsXTH16*, *RsXTH23*, *RsXTH24*, *RsXTH25*, and *RsXTH27*) were obviously upregulated exposed to Pb stress. However, five *RsXTH* genes (*RsXTH1*, *RsXTH2*, *RsXTH3*, *RsXTH4*, and *RsXTH5*) were notably downregulated in response to Pb stress (**Figures 4**, **5**). One possible explanation for the downregulation of these *RsXTH* genes is that it reflects a trade-off mechanism. Under Pb stress, plants may reallocate limited resources to prioritize essential survival processes, such as detoxification and antioxidant defense. Consequently, the expression of genes associated with growth and development, including certain *XTH* genes, is downregulated. This hypothesis is consistent with previous studies indicating that, during stress conditions, plants commonly suppress non-essential metabolic pathways to maintain vital physiological functions (Zhang et al., 2020a).

Although Pb stress can significantly induce changes in *XTH* expression and xyloglucan content, as reported in many studies (Gao et al., 2023; Zheng et al., 2021), there is still limited research on the specific biological functions of XTH in response to Pb stress. To the best of our knowledge, there is currently few reports showing that knocking out *XTH18* in *A. thaliana* significantly enhances Pb tolerance (Zheng et al., 2021). Here, we found that RsXTH25 positively regulated Pb stress response in *R. sativus* based on the following evidences: firstly, Pb stress obviously induced *RsXTH25* expression (**Figures 4**, **5**). Secondly, transiently overexpressing of *RsXTH25* significantly enhanced Pb tolerance (**Figures 6B–D**). Thirdly, transiently overexpression *RsXTH25* alleviated the oxidative damage caused by Pb stress (**Figures 6H, I**). Our study clearly confirmed the critical role of XTH in *R. sativus* response to Pb stress. However, it is worth noting that several other *RsXTH* genes were also induced by Pb stress, and whether they are involved in the response of *R. sativus* to Pb stress remains to be investigated in future studies.

In addition, our study successfully identified potential upstream transcription factors involved in the regulation of *RsXTH25* under lead (Pb) stress. Cluster analysis revealed that the gene module containing *RsXTH25* was enriched with multiple transcription factor families, including ERF and WRKY, which are known to play key roles in abiotic stress responses (Jiang et al., 2017; Wu et al., 2022). This suggests that these transcription factors may contribute synergistically to enhancing plant tolerance to Pb stress. Notably, transcription factors such as *RsERF2* and *RsHD-ZIP22* exhibited strong positive correlations with *RsXTH25* expression (**Figure 8**). It is hypothesized that these factors participate in Pb stress responses, such as cell wall remodeling and reactive oxygen species (ROS) scavenging, by directly binding to the *RsXTH25* promoter or indirectly regulating its expression via intermediate genes (Zhao et al., 2021). Future studies will be essential to validate these regulatory interactions and elucidate their biological functions, providing a theoretical foundation for the genetic improvement of Pb-tolerant crops.

In this study, phylogenetic analysis showed that 28 *RsXTH* genes were classified into five groups and distributed across 9 chromosomes. The analysis of conserved domains indicated a high similarity between RsXTHs and XTH proteins in other species. *RsXTH25* was significantly upregulated under Pb stress and positively regulates Pb stress response. Moreover, transcription factors such as *RsERF2* and *RsHD-ZIP22* showed strong positive correlations with *RsXTH25*, suggesting that they may function as upstream regulators mediating its expression under Pb stress.

## Data Availability

The original contributions presented in the study are included in the article/**Supplementary Material**. Further inquiries can be directed to the corresponding authors.

## Supplementary material

Supplementary Table 1 Primers used in this study.

Supplementary Table 2 The *XTH* genes list of *R. sativus*, *A. thaliana* and *O. sativa*.

Supplementary Table 3 Quality of transcriptome sequencing data.

Supplementary Table 4 List of differentially expressed genes under Pb stress.

Supplementary Table 5 The FPKM value of transcriptome data.
